# Development of a Simple 12-Item Theory-Based Instrument to Assess the Impact of Continuing Professional Development on Clinical Behavioral Intentions

**DOI:** 10.1371/journal.pone.0091013

**Published:** 2014-03-18

**Authors:** France Légaré, Francine Borduas, Adriana Freitas, André Jacques, Gaston Godin, Francesca Luconi, Jeremy Grimshaw, the CPD-KT team

**Affiliations:** 1 Public Health and Practice-Changing Research Group, CHUQ Research Centre (CRCHUQ), Quebec, Quebec, Canada; 2 Office of the Vice-Dean of Education and Continuing Professional Development, Faculty of Medicine, Université Laval, Quebec, Quebec, Canada; 3 Practice Enhancement Division, Collège des médecins du Québec, Montreal, Quebec, Canada; 4 Faculty of Nursing, Université Laval, Quebec, Quebec, Canada; 5 Continuing Health Professional Education Office, Faculty of Medicine, McGill University, Montreal, Quebec, Canada; 6 Clinical Epidemiology Program, Ottawa Hospital Research Institute, University of Ottawa, Ottawa, Ontario, Canada; 7 Department of Medicine, University of Ottawa, Ottawa, Ontario, Canada; University of Minho, Portugal

## Abstract

**Background:**

Decision-makers in organizations providing continuing professional development (CPD) have identified the need for routine assessment of its impact on practice. We sought to develop a theory-based instrument for evaluating the impact of CPD activities on health professionals' clinical behavioral intentions.

**Methods and Findings:**

Our multipronged study had four phases. 1) We systematically reviewed the literature for instruments that used socio-cognitive theories to assess healthcare professionals' clinically-oriented behavioral intentions and/or behaviors; we extracted items relating to the theoretical constructs of an integrated model of healthcare professionals' behaviors and removed duplicates. 2) A committee of researchers and CPD decision-makers selected a pool of items relevant to CPD. 3) An international group of experts (n = 70) reached consensus on the most relevant items using electronic Delphi surveys. 4) We created a preliminary instrument with the items found most relevant and assessed its factorial validity, internal consistency and reliability (weighted kappa) over a two-week period among 138 physicians attending a CPD activity. Out of 72 potentially relevant instruments, 47 were analyzed. Of the 1218 items extracted from these, 16% were discarded as improperly phrased and 70% discarded as duplicates. Mapping the remaining items onto the constructs of the integrated model of healthcare professionals' behaviors yielded a minimum of 18 and a maximum of 275 items per construct. The partnership committee retained 61 items covering all seven constructs. Two iterations of the Delphi process produced consensus on a provisional 40-item questionnaire. Exploratory factorial analysis following test-retest resulted in a 12-item questionnaire. Cronbach's coefficients for the constructs varied from 0.77 to 0.85.

**Conclusion:**

A 12-item theory-based instrument for assessing the impact of CPD activities on health professionals' clinical behavioral intentions showed adequate validity and reliability. Further studies could assess its responsiveness to behavior change following CPD activities and its capacity to predict health professionals' clinical performance.

## Introduction

Continuing professional development (CPD), including continuing medical education (CME) [1], is the method most commonly used by physicians to improve their knowledge and skills [2], [3]. However, in spite of physicians being regularly exposed to new research findings through attending CPD activities, incorporating new knowledge into professional practice is a slow process and delays patients' access to treatments of proven benefit [4]. In addition, producing and accrediting CPD events requires human resources, technology and other materials. In 2012, there were 1319 CME providers accredited by state medical societies in the US reporting expenses of $140.2 million for such activities [5]. It is vital to ensure that these resources constitute a worthwhile investment.

This study was initiated in 2009, when several decision-makers in CPD organizations in Canada formally met with knowledge translation (KT) researchers and identified the need for a short, user-friendly instrument that could be used to routinely assess the impact of CPD activities on clinical practice [6]. At the present time, most frameworks used to evaluate CPD are derived from Kirkpatrick's model [7], which assesses training effectiveness by measuring four distinct outcome levels: 1) participants' reactions to an educational activity; 2) participants' knowledge, skills, or attitudes; 3) transfer of learning to practice; and 4) the results of the newly acquired behavior on organizational outcomes such as productivity and quality. However, several CPD decision-makers in our milieu have noted that instruments for assessing the impact of CPD activities mostly focus on outcome levels 1 and 2, while outcome levels 3 and 4 are more important for CPD. They also expressed the need for an instrument that was theory-based, i.e. based on factors that are known to predict clinical behavior in health professionals.

A systematic review of 76 studies that used social-cognitive theories for explaining health professionals' clinical behavior [8] showed that the Theory of Planned Behavior (TPB) [9] was the most appropriate one for predicting health professionals' *behaviors*, while Triandis' theory [10] was a better model for explaining their *intentions to perform* the behaviors. A model that integrates these two well-known theories proposes that three categories of variables drive *behavior*: 1) behavioral intention (an individual's motivation) to adopt a specific behavior or not; 2) beliefs about one's capabilities (i.e. health professionals' perceptions of facilitators and barriers to adopting the behavior); and 3) habit (the frequency of performing the behavior in the past). The same integrated model of healthcare professionals' behaviors suggests that behavioral *intention* can be explained by: 1) beliefs about one's capabilities (also a determinant of behavior, as mentioned above); 2) beliefs about consequences; 3) moral norm (feeling of personal obligation regarding the adoption of the behavior); 4) social influences (perception of approval or disapproval by persons significant to the individual regarding the adoption of the behavior); 5) role and identity (beliefs about whether such behavior should be adopted by someone of similar age, sex or social position), and 6) characteristics of health professionals based on their socio-demographic data.

In the context of CPD, the constructs of the integrated model correspond to three of the four outcome levels proposed by Kirkpatrick's model. The Kirkpatrick Level 2 learning outcomes, attitude and skill improvements, relate to behavioral *intention*, whereas the Level 3 outcomes, transfer of learning to practice, relate to clinical *behavior*. The necessary transition from Level 2 to Level 3 can only occur as a result of changes promoted by the content and format of the CPD activity. This transition will depend on acquisition of skills, but also on other variables captured by the integrated model. The aim of the research project was to develop an instrument based on this integrated model to assess the impact of continuing professional development on clinical behavioral intentions [11]. This paper presents the first steps in the development of this instrument.

## Methods

A detailed protocol of this study has been published and is available on-line [11].

### Ethics Statement

Ethics approval for the project was received from the Research Ethics Board Committee of the Centre Hospitalier Universitaire de Québec (CHU de Québec) on 30 June 2010 (project # S10-06-033). Participants provided written informed consent to participate in this study. The ethics board committee approved the consent procedure used.

### Phase 1 – Systematic review

We updated an existing systematic review to October 31, 2010 [8]. Briefly, we sought instruments based on social-cognitive theories whose purpose was to predict healthcare professionals' clinically-oriented behavioral intentions and behaviors. Only instruments in English or French were included for item extraction. We extracted all items from eligible instruments and created an inventory of items. Pairs of reviewers independently analyzed all items to verify that they were adequately formulated to assess the constructs of the integrated model. Pairs of reviewers reclassified items when necessary to better match these constructs [8]. We computed the percentage of agreement between the authors' initial classifications and reviewers' findings on item phrasing. Discrepancies in reclassifications were resolved through discussion among team members. We removed duplicates by searching for common phrase structures to reduce the total number of items to be analyzed for each construct.

### Phase 2 – Selection of a preliminary set of items

Members of the research team formed a partnership committee of researchers and CPD decision-makers (n = 8) to select a preliminary set of items through a 3-step process. First, we provided members of the committee with the full list of items derived from Phase 1 for each of the constructs of the integrated model. Each member individually reviewed the inventory and ranked each item according to two criteria: a) how representative of the construct it was, and b) its applicability to different types of CPD activities. Second, AF produced a comprehensive list containing the most voted-for items for each of the constructs. Third, two conference calls were conducted to review the most voted-for items and to reach a consensus about which items should be included in a preliminary set of items.

### Phase 3 – E-Delphi study

#### Participants and recruitment strategy

An international group of experts was recruited; individuals were identified through team members' networks. To be eligible, they had to have expertise in knowledge translation, organization of CPD activities, clinical practice, measurement and evaluation, continuing medical education, or social-cognitive theories. They had to speak English or French (we conducted our e-Delphi in both languages).

#### Data collection and analysis

The process was conducted in a quasi-anonymous manner. Registered participants' emails were known only to the research coordinator to allow for sending reminders. Respondents' judgements and opinions remained strictly anonymous to other members of the expert group. For each round, participants were emailed a link to the questionnaire in the language of their choice (English or French), and were allotted two weeks to complete it. Email reminders were sent 48 hours before the deadline for each round.

The international group of experts was then asked to evaluate whether the items would be relevant for a generic tool that could be easily adapted to any CPD activity, using a 5-point Likert scale (1 = item completely irrelevant, 5 = item completely relevant). A clinical vignette illustrating how the proposed items could be used in a CPD activity whose learning objective was to perform a knee evaluation was given as an example, but participants were asked to rate their response to each item formulated in general terms (e.g. I intend to adopt the behavior described in the training activity objectives in my practice). In the first round, participants were asked simply to rate their responses to each item. In the second round, distributions of respondents' answers to each item in the previous round were presented in percentage form. In both rounds, participants were encouraged to comment both on the relevance of particular items and on the relevance of the questionnaire as a whole to evaluating the impact of the CPD activity on adoption of a clinical behavior. As there are no definite criteria for determining consensus in a Delphi study [12], content validity was set *a priori* when at least 75% of participants had reached agreement on the relevance of an item. A partial consensus was reached when more than 60% but less than 75% of participants agreed on an item's relevance. Absence of consensus was determined to be when less than 60% of participants agreed on the relevance of an item. Once the experts had completed this task, the partnership committee reviewed the final list of selected items. The committee analyzed the experts' comments on each item and reformulated the original items when judged necessary. Items that did not reach a consensus rate of at least 60% were excluded.

### Phase 4 – Assessment of the reliability and validity of the new theory-based instrument

#### Participants and recruitment strategy

The provisional instrument resulting from Phase 3 was assessed by participants recruited during a scientific conference at Université Laval, held in May, 2012. The conference included 20 lectures covering various topics related to emergency medicine, family medicine, palliative care, perinatology, and internal medicine. The target population was family physicians and residents. All participants who attended an eligible CPD activity were asked to complete the provisional questionnaire at the end of the activity and then again two weeks later. This resulted in recruitment of participants in 18 out of the 25 activities offered.

#### Data collection and analysis

Participants were asked to complete a self-administered paper-based questionnaire that was generated specifically for each CPD activity retained. The questionnaires were adapted by including one learning objective from the specific CPD activity in the wording of all items. Participants completed their questionnaire immediately after the CPD activity and again two weeks later. Statistical analysis was conducted on the healthcare professionals' evaluations of the items in such as a way as to preserve the structure of the integrated model, i.e. as all constructs had to have at least two items, the best scoring items for each construct were retained. Descriptive statistics including means, standard deviations, median and frequency were computed to summarize health professional characteristics and item responses for the test retest. Weighted kappa coefficients were calculated to assess test-retest reliability [13]. Exploratory factor analysis (EFA) was used to explore the factorial structure of the questionnaire. The number of factors was determined by three criteria: a) eigenvalues superior to 1; b) proportion of variance explained by the factor superior to 5%; and c) number of theoretical variables of the integrated model. Items were loaded if the load factor was ≥0.4. Cronbach alpha coefficients were calculated to assess internal consistency of each construct proposed by the EFA and to reduce the number of items in the questionnaire. A Cronbach alpha value ≥0.7 was considered acceptable. Then for each construct, missing values for the mean calculation were imputed if at least two items were available.

## Results

**Figure 1:**
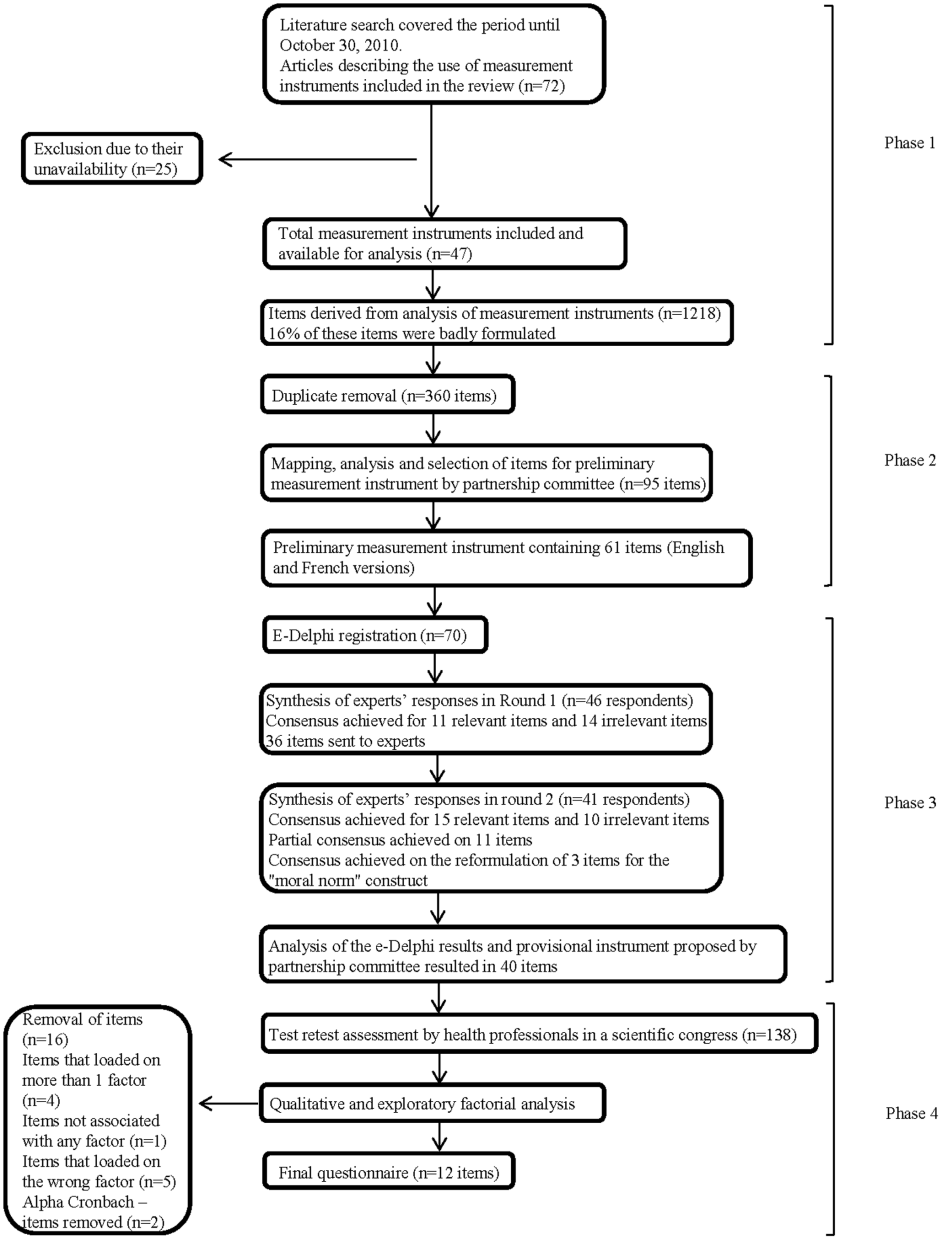
Flow chart describing the 4 phases of the development of the instrument.

Figure 1 shows the four phases in the development of the instrument.

### Phase 1 – Systematic review

We retrieved the 52 studies from the original systematic review [8] reporting good psychometric properties and identified 20 new studies from the updated searches covering October 30, 2007 to October 31, 2010 [14], [15], [16], [17], [18], [19], [20], [21], [22], [23], [24], [25], [26], [27], [28], [29], [30], [31], [32], [33]. Our review included 72 studies [14], [15], [16], [17], [18], [19], [20], [21], [22], [23], [24], [25], [26], [27], [28], [29], [30], [31], [32], [33], [34], [35], [36], [37], [38], [39], [40], [41], [42], [43], [44], [45], [46], [47], [48], [49], [50], [51], [52], [53], [54], [55], [56], [57], [58], [59], [60], [61], [62], [63], [64], [65], [66], [67], [68], [69], [70], [71], [72], [73], [74], [75], [76], [77], [78], [79], [80], [81], [82], [83], [84], [85]. We were able to get access to published versions or copies of 40 instruments [14], [15], [20], [21], [22], [23], [25], [26], [30], [31], [35], [37], [38], [41], [44], [45], [47], [48], [49], [51], [52], [53], [54], [55], [56], [57], [58], [60], [61], [64], [65], [67], [71], [73], [74], [76], [79], [83], [84], [85]. Authors of the 32 remaining tools [16], [17], [18], [19], [24], [27], [28], [29], [32], [33], [34], [36], [39], [40], [42], [43], [46], [50], [59], [62], [63], [66], [68], [69], [70], [72], [75], [77], [78], [80], [81], [82] were contacted and copies of their instruments requested. We received seven answers [29], [62], [63], [69], [72], [75], [80], resulting in 47 eligible instruments [14], [15], [20], [21], [22], [23], [25], [26], [29], [30], [31], [35], [37], [38], [41], [44], [45], [47], [48], [49], [51], [52], [53], [54], [55], [56], [57], [58], [60], [61], [62], [63], [64], [65], [67], [69], [71], [72], [73], [74], [75], [76], [79], [80], [83], [84], [85] that were included for data extraction. A total of 1218 items were identified. Among these, 194 items (16%) were not appropriately labeled in the published papers for measuring the constructs of the theoretical model used for instrument development. Overall, the percentage of agreement among reviewers on this assessment was 84%.

### Phase 2 – Selection of a preliminary set of items

Table 1 shows the results of each step in the generation of a preliminary set of items. First, the percentage of agreement among reviewers regarding mapping of the initial 1218 items onto constructs was 84%. Second, the removal of duplicates reduced the total number of items to 360 (see Table 1 for details). Third, members of the partnership committee individually analyzed each of the 360 generic items, and found only 95 items that met the two pre-established criteria, i.e. a) they were representative of one of the theoretical constructs of the integrated model and b) they could be adapted to any CPD context. The committee then re-analyzed these 95 items over two conference calls and selected 61 items to form a preliminary set that matched all the constructs.

**Table 1:** Item inventory and development of a preliminary set of items.

Constructs from the integrated model	N items extracted [references]	N items after duplicate removal	N items after individual analysis by partnership committee members	N items selected to be included in a preliminary set of items
**Intention**	**122** [14], [15], [16], [17], [18], [23], [24], [25], [26], [29], [32], [34], [38], [40], [41], [44], [47], [49], [50], [51], [54], [56], [57], [58], [59], [61], [62], [63], [64], [65], [66], [70], [72], [74], [75], [76], [77], [80], [81], [84]	39	11	6
**Beliefs about capabilities**	**275** [14], [15], [16], [17], [18], [23], [24], [25], [26], [28], [32], [34], [38], [40], [41], [44], [49], [50], [51], [53], [55], [56], [58], [61], [62], [65], [66], [68], [70], [72], [74], [77], [80], [81], [84]	113	15	12
**Habit/past behavior**	**15** [32], [44], [47], [51]	8	6	3
**Beliefs about consequences**	**466** [14], [15], [16], [17], [18], [23], [24], [25], [26], [28], [29], [32], [34], [40], [41], [44], [47], [49], [50], [51], [53], [55], [56], [57], [58], [59], [61], [62], [63], [64], [65], [66], [68], [70], [72], [73], [74], [75], [76], [77], [80], [81], [84], [85]	93	26	15
**Social influences**	**248** [16], [17], [18], [23], [24], [25], [26], [29], [32], [34], [40], [41], [44], [47], [49], [50], [51], [53], [55], [56], [57], [58], [61], [62], [63], [64], [65], [66], [68], [70], [72], [73], [74], [75], [76], [77], [80], [81], [84]	73	18	12
**Moral norm**	**18** [51], [55], [56], [64], [68], [80]	11	6	4
**Role and identity**	**33** [24], [32], [41], [44], [51], [56], [64], [65]	15	9	6
**Behavior**	**41** [15], [16], [25], [41], [56], [70], [84]	8	4	3
**Total**	**1218**	**360**	**95**	**61**

### Phase 3 – E-Delphi study

Seventy experts from different disciplines registered in the e-Delphi study (see Table 2). This group was composed equally of men and women. The majority of the experts registered in the study were French speakers (70%) and were from Canada (90%). Experts from the USA (n = 3), Australia (n = 2), Switzerland (n = 1) and The Netherlands (n = 1) also registered to participate. Table 3 summarizes the number of items per construct at each step of the e-Delphi study. Forty-six respondents participated in Round 1 (Figure 1). After synthesis of experts' responses, consensus was achieved among at least 75% of respondents that of the 61 items, 11 items were relevant and 14 items were irrelevant. These 25 items were excluded from Round 2 in order to focus on the items for which there was less clarity.

**Table 2:** Profile of international experts who participated in E-Delphi study (n = 70 registered participants).

Characteristics	N (%)
**Gender**	
Male	38 (54.3)
Female	32 (45.7)
**Age (years)**	
20–30	3 (4.3)
31–40	13 (18.6)
41–50	22 (31.4)
51–60	25 (35.7)
61 or more	7 (10)
**Area of expertise (not mutually exclusive)**	
Education	28 (40)
Clinical practice	34 (48.6)
CPD activities organization	35 (50)
Knowledge translation	36 (51.4)
Measurement and evaluation	21 (30)
Social-cognitive theories	14 (20)
**Language**	
French	49 (70)
English	21 (30)
**Country**	
Canada	63 (90)
USA	3 (4.3)
Australia	2 (2.9)
Switzerland	1 (1.4)
The Netherlands	1 (1.4)

**Table 3:** E-Delphi content validity process for design of provisional instrument.

Construct	Items/construct Preliminary set of items	e-Delphi Round 1			e-Delphi Round 2			Items for provisional questionnaire
		Items found relevant	Partial consensus	Items found irrelevant	Items found relevant	Partial consensus	Items found irrelevant	
**Beliefs about consequences**	15	5	6	4	3	1	2	**9**
**Beliefs about capabilities**	12	3	8	1	6	-	2	**9**
**Social influences**	12	-	5	7	1	4	-	**5**
**Intention**	8	3	3	2	2	-	1	**5**
**Role and identity**	4	-	4	-	1	3	-	**4**
**Moral norm**	4	-	4	-	1	-	3*	**4**
**Habit/past behavior**	3	-	3	-	1	1	1	**2**
**Behavior**	3	-	3	-	-	2	1	**2**
**Total**	**61**	**11**	**36**	**14**	**15**	**11**	**10**	**40**

* Items included after international expert group agreed on their reformulation.

For the second e-Delphi round, a 36-item questionnaire was sent back to the experts along with the mean scores for relevance obtained for each item at the first round. Forty-one respondents participated in Round 2 (Figure 1). After synthesis of the experts' responses, 15 further items were found relevant and 10 items were found irrelevant by at least 75% of respondents.

Taking both the integrity of the integrated model of healthcare professionals' behaviors and the results of the e-Delphi study into consideration, the partnership committee designed a 40-item provisional questionnaire which included 37 items that had achieved full (26) or partial (11) consensus after two rounds. In addition, there was consensus among the international experts that the three “moral norm” items, judged irrelevant in the e-Delphi process, would be relevant if formulated differently. Following their advice, the partnership committee rephrased these three items positively instead of negatively and reintegrated them into the provisional instrument. This resulted in a 40-item questionnaire with between two and ten items for each construct: beliefs about consequences (n = 9), beliefs about capabilities (n = 9), social influences (n = 5), intention (n = 5), role and identity (n = 4), moral norm (n = 4), habit/past behavior (n = 2), and behavior (n = 2).

### Phase 4 – Assessment of the reliability and validity of the new theory-based instrument

#### Characteristics of healthcare professionals included in the study

Table 4 shows the characteristics of the 138 participants included in this phase of the study, i.e. healthcare professionals who were engaged in CPD activities at a scientific congress and who filled out questionnaires (test). Of the 138 participants, 76.8% were female. Among the participants, 42% were residents, 49.3% were family physicians, 2.2% were specialists, and 6.5% were healthcare professionals other than physicians. The same questionnaire was returned two weeks later (retest) by 88% of participants (121/138). In contrast with the procedure described in the study protocol [11], the partnership committee reviewed the preliminary analysis based on the items validated in the e-Delphi study and removed 16 out the 40 items to respect the principles of the theoretical model. Specifically, the items concerning the construct “habit” were removed because they did not reflect the model's definition of habit (poor wording).

**Table 4:** Characteristics of participants in the test-retest of the CPD assessment tool (n = 138).

Characteristics	N (%)
**Age (years)**	
20–30	58 (42)
31–40	43 (31.2)
41–50	24 (17.4)
51–60	13 (9.4)
**Gender**	
Female	106 (76.8)
**Professional status**	
Resident	58 (42)
Family physician	68 (49.3)
Specialist	3 (2.2)

#### Test retest reliability

The first data collection in this phase and the second data collection two weeks later provided data for assessing the reliability of the instrument over time. Test-retest reliability was moderate (Table 5) with weighted kappa values between 0.4 and 0.6.

**Table 5:** Test-retest reliability for the items in the final CPD assessment tool (n = 138 for test and n = 121 for retest).

Item	Test			Retest			Reliability (weighted kappa)
	N	Mean (sd)	Median (min-max)	N	Mean (sd)	Median (min-max)	
**Q1**	137	6.14 (1.28)	7 (1–7)	121	6.02 (1.27)	6 (2–7)	0.54
**Q2**	137	6.26 (1.28)	7 (1–7)	121	6.17 (1.16)	7 (2–7)	0.56
**Q4**	137	5.55 (1.55)	6 (1–7)	121	5.55 (1.43)	6 (2–7)	0.51
**Q5**	136	4.23 (1.54)	5 (1–6.5)	121	4.22 (1.52)	5 (1–6.5)	0.45
**Q7**	138	5.38 (1.22)	5 (1–7)	121	5.60 (1.14)	6 (2–7)	0.53
**Q8**	138	5.54 (1.12)	6 (2–7)	120	5.57 (1.17)	6 (2–7)	0.41
**Q10**	137	5.13 (1.38)	5 (1–7)	121	5.27 (1.18)	5 (2–7)	0.42
**Q12**	137	6.14 (1.18)	7 (1–7)	121	5.99 (1.18)	6 (2–7)	0.60
**Q13**	138	6.35 (0.97)	7 (1–7)	121	6.32 (0.93)	7 (2–7)	0.52
**Q15**	138	3.43 (1.87)	3 (1–7)	121	3.26 (1.79)	3 (1–7)	0.41
**Q16**	138	6.21 (1.14)	7 (1–7)	121	6.13 (1.21)	7 (1–7)	0.59
**Q18**	138	6.01 (1.22)	6 (1–7)	121	6.03 (1.10)	6 (1–7)	0.50
**Q21**	137	5.32 (1.61)	6 (1–7)	120	5.14 (1.58)	5 (1–7)	0.50
**Q22**	138	4.11 (2.08)	4 (1–7)	121	4.41 (2.03)	5 (1–7)	0.56
**Q23**	138	6.41 (0.93)	7 (2–7)	121	6.26 (0.99)	7 (2–7)	0.55
**Q25**	138	5.86 (1.16)	6 (1–7)	121	5.82 (1.22)	6 (1–7)	0.43
**Q26**	138	6.14 (1.10)	6 (1–7)	121	6.07 (1.11)	6 (1–7)	0.49
**Q28**	137	5.31 (1.25)	5 (1–7)	121	5.39 (1.33)	6 (1–7)	0.53
**Q29**	138	6.64 (0.65)	7 (4–7)	121	6.44 (0.81)	7 (3–7)	0.45
**Q30**	138	5.12 (1.51)	5 (1–7)	121	5.07 (1.58)	5 (1–7)	0.53
**Q31**	138	4.89 (1.11)	5 (2–7)	121	4.96 (1.12)	5 (2–7)	0.48
**Q32**	138	6.49 (0.79)	7 (4–7)	121	6.36 (0.82)	7 (4–7)	0.42
**Q35c**	138	6.46 (0.94)	7 (2–7)	121	6.48 (0.87)	7 (3–7)	0.50
**Q35d**	138	6.55 (0.76)	7 (4–7)	120	6.43 (0.84)	7 (4–7)	0.54

#### Exploratory factorial analysis (EFA)

EFA showed that four items loaded on more than one construct and they were therefore excluded. One item did not load on any construct and five items loaded on the wrong constructs, leaving 14 remaining items. All factors had eigenvalues superior to 1; the proportion of variance explained by the factor was superior to 5%. During this process, the items relating to the construct “role and identity” were removed because no items loaded on any factors as defined by EFA.

#### Cronbach alpha coefficients

Cronbach alpha coefficients were considered acceptable (range: 0.79 to 0.89). Two items were removed because of poor consistency (Cronbach alpha <0.70), resulting in a final questionnaire of 12 items (Table 6).

**Table 6:** Exploratory factorial analysis (12-item final instrument).

Item		Factor 1 (Beliefs about capabilities)	Factor 2 (Social influences)	Factor 3 (Beliefs about consequences)	Factor 4 (Moral norm)	Factor 5 (Intention)
**Q7**	I have the ability to [*behavior*] (strongly disagree/strongly agree)	**0.74934**	0.18021	−0.12599	0.21059	−0.05858
**Q25**	I am confident that I could [*behavior*] (strongly disagree/strongly agree)	**0.78329**	−0.09811	0.08429	0.03143	0.18647
**Q31**	For me, [*behavior*] would be (extremely difficult/extremely easy)	**0.96592**	−0.03217	0.04087	−0.17871	0.02051
**Q5**	To the best of my knowledge, the proportion of colleagues who will [*behavior*] would be: (0–100%)	−0.03024	**0.81163**	−0.04977	−0.14544	0.21287
**Q8**	Now think about a co-worker who you respect as a professional. In your opinion, does he/she [*behavior*] (never/always)	0.14637	**0.87245**	−0.04222	0.13894	−0.21688
**Q10**	Most persons who are important for me in the profession would [*behavior*] (strongly disagree/strongly agree)	−0.11913	**0.74838**	0.16943	−0.06198	0.22519
**Q35c**	Overall, I think that [*behavior*] is, for me: (useless/useful)	0.05487	−0.04288	**0.94033**	−0.01561	0.01417
**Q35d**	Overall, I think that is [*behavior*]: (harmful/beneficial)	−0.04608	0.06134	**0.93328**	0.05920	−0.04677
**Q29**	[*Behavior*] is the ethical thing to do (strongly disagree/strongly agree).	−0.07456	−0.10323	−0.08101	**0.93819**	0.17571
**Q32**	It would be acceptable to [*behavior*] (strongly disagree/strongly agree)	0.04682	0.05495	0.22567	**0.77132**	−0.03758
**Q1**	I intend to [*behavior*] (strongly disagree/strongly agree)	0.00976	0.12708	−0.09372	0.12053	**0.83805**
**Q12**	I plan to [*behavior*] (strongly disagree/strongly agree)	0.22201	0.01258	0.14262	0.07211	**0.66377**
	**Variability (%)**	45.5	12.4	10.4	8.1	5.4
	**Eigenvalues**	5.5	1.5	1.3	1	0.6
	**Cronbach alpha**	0.84	0.79	0.89	0.8	0.79

## Discussion

This study established the reliability and validity of a simple 12-item theory-based instrument for evaluating the impact of CPD activities on health professionals' clinical behavioral intentions. Based on an extensive systematic review, we found a total of 1218 items from 47 eligible instruments. Sixteen per cent were not appropriately labeled and most were duplicates, leaving us with 360 original items covering the theoretical constructs of the integrated model of healthcare professionals' behaviors. In a second phase, members of a partnership committee selected 61 of these 360 items based on how well they represented one of the theoretical constructs of interest and on their adaptability to any CPD context. Then, through an e-Delphi process, an international group of experts from a wide range of disciplines achieved consensus on 37 items which were included in a first version of a 40-item questionnaire. In a last phase, 138 physicians helped establish that the final 12-item instrument had validity including adequate factorial structure and reliability. These results lead us to make four main observations.

First, during our initial phase we found that a significant proportion of the items derived from the systematic review, i.e. 16%, were not appropriately formulated to measure the constructs of the theories the authors had used to develop their tools, but applied instead to other constructs. This may have created a bias in the findings of the authors we consulted regarding the relationship between one given variable (e.g. attitude of health professionals) and another (e.g. clinical behavior). While these authors often associated the weakness of their results with the absence of validation procedures for their questionnaires [21], [29], [30], they rarely questioned if their poor results were a consequence of inappropriate wording of items in relation to the theory on which the questionnaires were based [28]. The majority of items, moreover, although they had slight variations in wording, were found to be duplicates. These observations clearly indicate that there is a need for a theory-based instrument that is validated and reliable for assessing the impact of CPD activities on health professional behaviors. This will help standardize the presentation of the many factors that are likely to influence the uptake of new clinical behaviors [86]. In turn this will: 1) facilitate comparisons between similar studies, 2) make it possible to carry out a systematic review in this area, 3) help inform policy makers about how to change clinical behaviors and, most importantly, 4) contribute to the elaboration of a theoretical base for translating evidence into clinical practice.

Second, in subsequent phases of this project, it became clear that theory-based items used for studying healthcare professionals' behaviors were not all relevant or applicable to the CPD context. CPD developers who had initiated this project had made very clear that they wanted a short, simple instrument that could be easily disseminated across the CPD environment. Their difficulties in this regard are consistent with those reported by implementation scientists trying to assess the impact of their interventions with busy clinicians. The proposed instrument is based on well-validated socio-cognitive theories, and yet includes only 12 items. It may therefore contribute not only to a more theory-based approach to CPD assessment but to a more pragmatic one.

Third, the e-Delphi study asked the international experts to evaluate items in generic terms, but to facilitate comprehension we presented an example extracted from a clinical vignette used in a specific CPD activity (knee evaluation). This may explain why the majority of the international experts judged that the items relating to “moral norm” were not relevant when negatively formulated. However, in the last phase of this study we demonstrated that the instrument can be easily adapted to different clinical areas of medicine. When we tested the instrument with participants in a scientific congress, we observed that the items adapted well to a variety of clinical areas such as emergency medicine, family medicine, palliative care, perinatology, and internal medicine. Adapting the tool to each context simply requires rewording each of the 12 items so that they include the observable behavior described in the learning objective of the activity. Indeed, the generic and adaptable nature of the instrument to a wide range of clinical domains is its main advantage over other such instruments [87].

Lastly, in order to develop this tool, our multidisciplinary partnership committee used a highly systematic multi-phase approach to refine the link between the items and the theoretical constructs they are supposed to measure. Although designing CPD strategies that will impact health professionals' behavior and improve patient outcomes is complex, effective CPD is an essential vehicle for knowledge translation [88]. During our whole project, a strong partnership between researchers and CPD developers ensured that all concerns would be taken into account so that our final instrument would prove useful to both educators and researchers. We believe that the newly developed instrument will not only help CPD developers improve the effectiveness of their training programs by paying more attention to the socio-cognitive factors known to predispose healthcare professionals to change their clinical behavior, but also help researchers who are interested in further exploring these socio-cognitive factors.

This study has some limitations. Studies reporting the development of instruments are generally not well-indexed in electronic databases [89]. Some eligible instruments may not have been captured by the search strategy used. Our analysis was also restricted to instruments of which it was possible to obtain a copy. However, given the large number of duplicates among the items found, we are confident that finding additional instruments would not have had an impact on our study results. In this study, we accepted the premise that behavioral intention is an immediate antecedent of behavior, and that intention provides a proxy measure of physicians' behavior [9] that corresponds to Kirkpatrick's Level 3 [7]. Nonetheless, further studies are necessary to demonstrate a correlation between behavioral intention and observed behavior of healthcare professionals subsequent to CPD interventions.

## Conclusion

In conclusion, we believe that this new 12-item theory-based instrument with robust metric properties is appropriate for the routine assessment of the impact of CPD activities on clinical behavioral intention change. We plan further studies to validate on a larger scale its ability to predict behavior and its sensitivity to change in response to CPD activities. We will also verify the acceptability of the 12-item instrument among CPD providers by examining the barriers and facilitating factors associated with the implementation of the new tool in the province of Quebec, Canada.
